# The Relationship between Exercise Self-Efficacy, Intention, and Structural Barriers for Physical Activity after a Cancer Diagnosis

**DOI:** 10.3390/cancers14102480

**Published:** 2022-05-18

**Authors:** Johanna Depenbusch, Alexander Haussmann, Joachim Wiskemann, Angeliki Tsiouris, Laura Schmidt, Monika Sieverding, Nadine Ungar, Karen Steindorf

**Affiliations:** 1 Division of Physical Activity, Prevention and Cancer, National Center for Tumor Diseases (NCT) Heidelberg and German Cancer Research Center (DKFZ), Im Neuenheimer Feld 581, 69120 Heidelberg, Germany; johanna.depenbusch@nct-heidelberg.de (J.D.); alexander.haussmann@nct-heidelberg.de (A.H.); 2 Medical Faculty, Heidelberg University, Im Neuenheimer Feld 672, 69120 Heidelberg, Germany; 3 Division of Medical Oncology, National Center for Tumor Diseases (NCT) Heidelberg and University Clinic Heidelberg, Im Neuenheimer Feld 460, 69120 Heidelberg, Germany; joachim.wiskemann@nct-heidelberg.de (J.W.); angeliki.tsiouris@unimedizin-mainz.de (A.T.); 4 Department of Psychosomatic Medicine and Psychotherapy, University Medical Center Mainz, Johannes Gutenberg University Mainz, Untere Zahlbacher Straße 8, 55131 Mainz, Germany; 5 Institute of Psychology, Heidelberg University, Hauptstraße 47-51, 69117 Heidelberg, Germany; laura.schmidt@psychologie.uni-heidelberg.de (L.S.); monika.sieverding@psychologie.uni-heidelberg.de (M.S.); nadine.ungar@psychologie.uni-heidelberg.de (N.U.); 6 Faculty of Healthcare and Nursing, Catholic University of Applied Sciences Mainz, Saarstraße 3, 55122 Mainz, Germany

**Keywords:** cancer, exercise oncology, impediment, intention, physical activity, structural barriers, self-efficacy

## Abstract

**Simple Summary:**

Despite numerous benefits of physical activity for cancer patients, the majority is insufficiently active. Previous research has shown that structural barriers negatively affect patients’ physical activity behavior. Identifying underlying mechanisms could help to develop effective strategies that alleviate those barriers and increase physical activity levels. In the current survey study, we investigated whether cancer patients’ self-efficacy, i.e., their confidence in their ability, and their intention to exercise mediated the relationship between structural barriers and physical activity. The results revealed a negative relation between structural barriers and patients’ self-efficacy. Lower self-efficacy, in turn, decreased patients’ intention and their likelihood to engage in physical activity. This mediating effect especially applied to those individuals who were sufficiently active before the diagnosis. Thus, the findings suggest that interventions directly addressing the perception of structural barriers or patients’ self-efficacy in dealing with these barriers might be effective in improving the physical activity levels of cancer patients.

**Abstract:**

Previous research has shown that structural barriers negatively influence the physical activity (PA) behavior of cancer patients, but underlying mechanisms are unclear. The aim of the current study was to explore the potential mediating role of social-cognitive factors, namely PA self-efficacy and PA intention in this context. A total of 856 cancer patients completed a questionnaire on sociodemographic and medical characteristics, pre- and post-diagnosis PA, PA self-efficacy, PA intention, and PA impediment by structural barriers. A serial mediation model was used to test whether the association between structural barriers and post-diagnosis PA was mediated by PA self-efficacy and/or PA intention, in the overall sample and in subsamples defined by individuals’ pre-diagnosis PA. The results confirmed that structural barriers were not directly (95%CI [−0.45; 0.10]) but indirectly associated with post-diagnosis PA. Higher impediment by structural barriers decreased the likelihood of sufficient post-diagnosis PA via lower PA self-efficacy (95%CI [−0.25; −0.06]) and via the serial pathway of lower PA self-efficacy and lower PA intention (95%CI [−0.19; −0.05]). Investigating differences in these mediations by pre-diagnosis PA yielded significance only among previously active cancer patients. Both structural barriers and PA self-efficacy might hence be relevant target points for interventions aiming to improve PA behavior, especially among pre-diagnosis active cancer patients.

## 1. Introduction

The benefits of physical activity (PA) for the physical and mental health of people with cancer are widely acknowledged. Previous research has demonstrated that PA is not only associated with increased physical functioning and fitness [1,2], reduced treatment-related side effects [3,4], and improved quality of life [2], but it might also be beneficial to reducing the risk of cancer-specific mortality and recurrence [5]. Based on the evidence about the safety and effectiveness of PA during and after cancer treatment, current recommendations state that people with cancer should aim towards at least 30 min of aerobic activities three times per week and include resistance exercises twice per week [6]. However, the physical and structural changes and emotional distress associated with cancer diagnosis and treatment exacerbate the initiation and maintenance of PA and thus the compliance with recommended guidelines. As past studies indeed reported insufficient PA levels for the majority of cancer patients [7,8,9], effective strategies are needed to counteract the challenges that prevent cancer patients from exercising. A number of studies have elaborated on the role of disease- and treatment-related barriers in this regard [10,11,12,13]. However, it appears reasonable to also have a closer look at structural barriers, such as lack of information material or suitable exercise facilities, since these barriers have been suggested to markedly decrease the likelihood of reaching sufficient activity levels post-diagnosis [14,15,16].

A better understanding of the mechanisms through which structural barriers impede the activity behavior of people with cancer could help to identify target points for successful interventions. In this context, social-cognitive factors may play an important role as they are not only considered highly relevant for the initiation and maintenance of PA behavior but are also amenable to interventions [17]. Many widely acknowledged social-cognitive theories that aim to explain PA behavior include PA self-efficacy and PA intention as key constructs. PA self-efficacy is defined as the individual’s confidence in his/her ability to perform PA despite potential barriers, while PA intention describes the individual’s motivation and determination to exercise [18,19,20]. The relationship between PA self-efficacy and PA intention and their association with PA behavior have been extensively elaborated in prior research, also among people with cancer [21]. The results demonstrated that cancer patients’ PA self-efficacy was a strong predictor of their intention to exercise, which in turn was consistently revealed as a significant determinant of PA behavior after the cancer diagnosis [22,23,24,25,26,27,28]. Additionally, direct associations were detected between PA self-efficacy and post-diagnosis PA [23,28,29]. Their influential relationship with PA among people with cancer was further strengthened by a recent systematic review and meta-analysis with meta-analytic path modeling. The results showed an excellent fit for a model including a direct path from PA intention to PA behavior as well as the two suggested paths from PA self-efficacy to PA behavior, i.e., direct and indirectly via PA intention [21].

It is conceivable that cancer patients’ confidence and motivation to engage in PA depend on whether the exercise environment and infrastructure support the specific needs of this target group. Thus, a perceived lack of essential structural preconditions that enable PA during as well as after cancer treatment could negatively affect the individual’s self-efficacy and intention to exercise. Previous studies among patients with chronic diseases, including breast cancer, suggested that the perception of barriers was significantly negatively associated with PA self-efficacy, which in turn was directly related to PA behavior [30,31,32]. The mediating role of PA intention in this relationship has, however, been neglected so far. The definition of PA barriers in the above-cited studies further comprised a broader range of impeding factors so that the particular relationship between structural barriers and social-cognitive constructs remains unclear. In order to inform strategies for cancer patients to overcome structural barriers to PA, it thus appears meaningful to explore the role of PA self-efficacy and PA intention for the association between structural barriers and sufficient PA after the cancer diagnosis. As not only sufficient post-diagnosis PA [7,9,22,33,34] but also its association with structural barriers [16] were shown to differ between pre-diagnosis active and insufficiently active individuals, a distinction between these two subgroups could shed further light on how to specifically target interventions addressing structural PA barriers.

Thus, the aims of the present study were to (a) investigate whether PA self-efficacy and PA intention serve as mediating factors for the association between structural barriers and sufficient PA after the cancer diagnosis and (b) explore whether the proposed mediation model equally applies to the maintenance of PA levels among previously sufficiently active individuals and the increase in PA levels among previously insufficiently active individuals.

## 2. Materials and Methods

### 2.1. Design and Participants

The present cross-sectional study was part of the large-scale Momentum project Heidelberg, which has been registered under NCT02678832 at clinicaltrials.gov. The survey study focused on social cognitions and norms regarding PA among people with cancer and was conducted between January 2017 and May 2018. Eligible were individuals ≥18 years with breast, prostate, or colorectal cancer whose latest diagnosis of the primary tumor, recurrence, or metastases was no longer than 30 months ago. This target group was addressed since breast, prostate, and colorectal cancer constitute prevailing cancer types, for which the beneficial effects of PA during and shortly after cancer treatment have been extensively investigated [35,36]. Further, participants had to either have completed cancer treatment within the previous 30 months, been currently undergoing it, or planned to receive cancer treatment, i.e., surgery, chemotherapy, and/or radiation therapy. Last, they were required to be capable of standing and walking without assistive devices. Recruitment of participants was mainly carried out via the cancer registry in Baden-Württemberg. Additional recruitment strategies involved physicians, who took part in the Momentum healthcare professional study, as well as information events, self-help group associations, and online portals for cancer patients. Further details on recruitment and study conduct have been reported elsewhere [9]. The Faculty of Behavioral and Cultural Studies of Heidelberg University provided ethical approval [AZ Siev 2015/1-1, AZ Siev 2016/1-2], and all participants signed informed consent forms.

### 2.2. Measures

All information was assessed in a paper-and-pencil or congruent online survey as self-reported data by participants. Sociodemographic and medical items included age, sex, educational degree, height and weight to determine the body mass index (BMI), cancer type and the corresponding date of diagnosis, type and status of cancer treatment, and co-morbidities based on the Charlson co-morbidity index [37]. Perception of PA counseling by physicians was inquired according to the 5A framework [38] and summarized as a weighted counseling score (5A score) with higher values indicating more comprehensive exercise counseling [39].

PA assessment was adapted from the Godin-Shepard Leisure-Time Physical Activity Questionnaire [40]. Participants were asked to estimate their average weekly minutes of light, moderate, and vigorous physical activities pre-diagnosis and post-diagnosis, i.e., within the last week. For each timepoint, moderate- and two times vigorous-intensity PA minutes were summed as an indicator of weekly minutes of moderate-to-vigorous PA (MVPA). In accordance with recommended guidelines at that time [41], a cut point of 150 min MVPA per week was chosen to classify individuals as either sufficiently active (≥150 min MVPA/week) or insufficiently active (<150 min MVPA/week) for both timepoints.

PA impediment by structural barriers was inquired with seven items that were developed from our preceding qualitative and quantitative study among healthcare professionals [42,43]. Each item asked to what extent the respective factor prevented the participants from regularly performing PA: (1) lack of information material regarding PA for cancer patients, (2) lack of PA offers specifically for cancer patients, (3) lack of PA offers overall, (4) lack of possibility to clarify if one is medically suitable for PA, (5) lack of an expert contact person, (6) lack of therapeutic programs that are reimbursed by healthcare insurances, and (7) lack of parks, walking, running, and cycling paths, or public pools in the neighborhood [16]. Answer options ranged from 0 ‘not at all’ to 3 ‘very strongly’. A mean barrier score was calculated from the total sum of all seven barriers. The intention to be sufficiently active was measured with two items. First, participants were asked to indicate whether they intended to perform at least 150 min of at least moderate-intensity PA in the upcoming three months on a 7-point Likert scale from 0 ‘no, not at all’ to 6 ‘yes, definitely’. Subsequently, participants estimated their likelihood of performing at least 150 min of at least moderate-intensity PA in the upcoming three months in percent. This percentage was multiplied by 6 and divided by 100 to obtain the same scaling for both items and thus enable the calculation of a mean intention value. For PA self-efficacy, participants rated on a 7-point Likert scale to what extent the statements ‘I find it easy to be at least moderately physically active for at least 150 min per week’ and ‘I am confident that I would be able to be at least moderately physically active for at least 150 min per week’ applied to them. The two PA self-efficacy items were also averaged.

### 2.3. Statistical Analyses

All statistical analyses were conducted with IBM SPSS version 25. Descriptive statistics (means (*M*) and standard deviations (*SD*) for metric variables and counts and percentages for nonmetric variables) were used to determine sociodemographic and medical characteristics of the study sample, their pre- and post-diagnosis PA behavior, perceived PA impediment by structural barriers, as well as their self-efficacy and intention to be physically active. Pearson’s correlation coefficient *r* and point biserial correlations (*r*_pb_) were calculated to ensure significant bivariate correlations between the main variables of the following mediation analysis.

**Figure 1:**
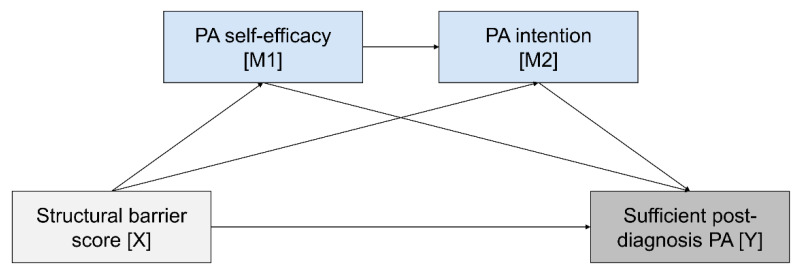
Conceptual research model with the effect of structural barriers on post-diagnosis physical activity being mediated through social-cognitive factors.

To determine the relationship between the structural barrier score, PA self-efficacy, PA intention, and sufficient post-diagnosis PA, a serial mediation model was tested using the PROCESS macro for SPSS (v.4.0). The structural barrier score served as the predictor (X), PA self-efficacy as the first mediator (M1), PA intention as the second mediator (M2), and meeting PA guidelines post-diagnosis as the outcome variable (Y) (Figure 1).

As such serial mediation model with two mediators comprises three criterion variables (M1, M2, and Y), direct associations with their specified predictors can be determined in three separate regression analyses. Accordingly, in the current model, the first regression analysis examined the association of the first mediator PA self-efficacy with its antecedent structural barrier score, the second one tested the associations of the second mediator PA intention with its antecedents PA self-efficacy and structural barrier score, and the third one analyzed the associations of the outcome sufficient post-diagnosis PA with its antecedents PA intention, PA self-efficacy, and structural barrier score.

Finally, the hypothesized pathways for direct and indirect effects in the full serial mediation model were evaluated. The applied model allowed to probe three mediation pathways for indirect effects: (a) the indirect effect of the structural barrier score on sufficient post-diagnosis PA through PA self-efficacy (X → M1 → Y), (b) the indirect effect of the structural barrier score on sufficient post-diagnosis PA through PA intention (X → M2 → Y), and (c) the indirect effect of the structural barrier score on sufficient post-diagnosis PA through PA self-efficacy and PA intention in serial (X → M1 → M2 → Y). The analyses were adjusted for age, sex, and sociodemographic, medical, and behavioral covariates that were shown to be significantly associated with sufficient post-diagnosis PA in the same sample, i.e., educational level, BMI, cancer type, time since latest diagnosis, co-morbidities, 5A score, and sufficient pre-diagnosis PA [9,39]. Estimated direct and indirect effects were probed by generating 10,000 bootstrapped samples and appraised with 95% confidence intervals (CI).

Following the mediation analysis in the overall sample, the same procedure was applied separately in subsamples defined by whether or not participants were meeting PA guidelines pre-diagnosis to explore potential differences in the proposed associations between previously sufficiently and previously insufficiently active individuals.

## 3. Results

### 3.1. Descriptive Statistics and Bivariate Correlation Analyses

**Figure 2:**
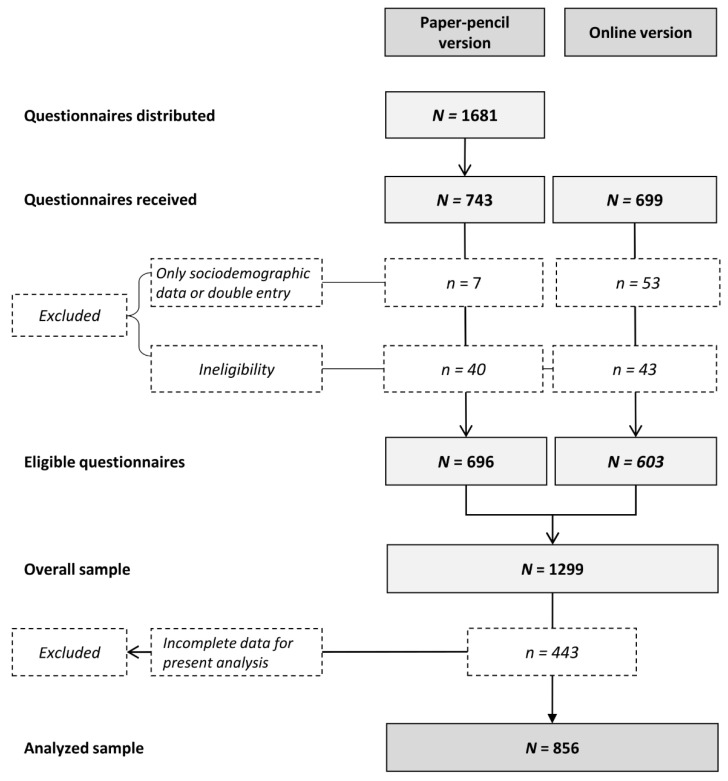
Recruitment flow. Adapted from ‘Change patterns and determinants of physical activity differ between breast, prostate and colorectal cancer patients,’ by K. Steindorf, 2020, *Support Care Cancer* [9]. Copyright 2019 by Springer-Verlag GmbH Germany, part of Springer Nature.

The overall sample consisted of 1299 people with cancer, of which 856 provided complete information on the variables included in the current analyses (Figure 2). Participants were, on average, 58.2 years old (*SD* = 12.4), and 60% were female. Half of the participants were diagnosed with breast (51%) and about one-quarter with either prostate (25%) or colorectal cancer (24%). The mean time since diagnosis was 14.8 months (*SD* = 7.6), and 45% of patients were receiving treatment at the time of study completion (Table 1). PA measures yielded that the proportion of sufficiently active individuals descriptively decreased from 63% pre-diagnosis to 55% post-diagnosis. Looking at social-cognitive constructs, participants indicated an average value of 4.9 out of 6 (*SD* = 1.3) for their intention to perform 150 min MVPA per week and an average value of 4.4 out of 6 (*SD* = 1.7) for their self-efficacy to be sufficiently active. The mean structural barrier score describing the perceived impediment by seven structural barriers was 0.7 (*SD* = 0.7). Bivariate correlation analyses revealed that the structural barrier score was negatively correlated with PA self-efficacy (*r* = −0.224, *p* < 0.001), PA intention (*r* = −0.175, *p* < 0.001), and sufficient post-diagnosis PA (*r*_pb_ = −0.148, *p* < 0.001). The correlation matrix further showed a strong correlation between PA self-efficacy and PA intention (*r* = 0.744, *p* < 0.001) as well as medium-sized correlations of both PA self-efficacy (*r*_pb_ = 0.470, *p* < 0.001) and PA intention (*r*_pb_ = 0.469, *p* < 0.001) with sufficient post-diagnosis PA.

### 3.2. Mediation Model

Results of the separate regression analyses testing the proposed associations within the mediation model are displayed in Table 2. The analyses yielded that the structural barrier score was significantly negatively associated with PA self-efficacy (*p* < 0.001, 95% CI [−0.53; −0.17]), but not with PA intention (*p* = 0.630, 95% CI [−0.13; 0.08]) or sufficient post-diagnosis PA (*p* = 0.171, 95% CI [−0.47; 0.08]). PA self-efficacy turned out as the strongest predictor of PA intention (*p* < 0.001, 95% CI [0.50; 0.60]) and was further related to sufficient post-diagnosis PA (*p* < 0.001, 95% CI [0.25; 0.57]). Additionally, PA intention emerged as a significant predictor of sufficient post-diagnosis PA (*p* < 0.001, 95% CI [0.33; 0.78]).

Table 3 shows the results of the actual mediation analysis, probing the direct and three possible indirect effects of the structural barrier score on sufficient post-diagnosis PA via the suggested mediators PA self-efficacy and PA intention. The analyses revealed that the structural barrier score was not directly (95% CI [−0.47; 0.08]) but indeed significantly indirectly associated with sufficient post-diagnosis PA (Boot 95% CI [−0.42; −0.12]), with two of the three possible mediation pathways being statistically significant. Firstly, the effect of the structural barrier score on post-diagnosis PA was mediated by PA self-efficacy (Boot 95% CI [−0.25; −0.06]), in the sense that a higher barrier score was significantly associated with lower PA self-efficacy, which in turn decreased the likelihood of being sufficiently active post-diagnosis. While the indirect effect of the structural barrier score via intention on sufficient post-diagnosis PA did not turn out statistically significant (Boot 95% CI [(−0.08; 0.04]), the analyses confirmed the serial mediation through both mediators PA self-efficacy and PA intention (Boot 95% CI [−0.19; −0.05]): Participants who reported stronger structural barriers indicated a lower PA self-efficacy, which in turn was associated with decreased PA intention. This again decreased the likelihood of being sufficiently active post-diagnosis.

### 3.3. Subgroup Analyses by Pre-Diagnosis PA

Analyzing the direct and indirect effects of the proposed mediation model in subgroups defined by whether or not participants were meeting PA guidelines before the diagnosis revealed that the effects indeed differed depending on patients’ pre-diagnosis PA (Table 3). While there was no direct association of the structural barrier score with sufficient post-diagnosis PA in either of the subgroups, significant indirect effects could be detected for the subgroup of previously sufficiently active individuals. Equivalent to the analyses in the overall sample, the effect of the structural barrier score on the maintenance of sufficient PA levels was mediated by PA self-efficacy (95% CI [−0.41; −0.10]) as well as via the serial pathway, including both mediators PA self-efficacy and PA intention (95% CI [−0.22; −0.03]). In contrast, none of the mediation pathways turned out statistically significant for the subgroup of previously insufficiently active individuals.

## 4. Discussion

The current study corroborated the mediating role of cancer patients’ self-efficacy and intention to exercise for the association between structural barriers and sufficient PA levels after the cancer diagnosis. The results indicated that a higher impediment by structural barriers was significantly associated with lower PA self-efficacy but did not directly influence the intention to exercise or activity behavior itself. As proposed, the effect of structural barriers on sufficient post-diagnosis PA was mediated through PA self-efficacy as well as a serial mediation pathway via both PA self-efficacy and PA intention. Subgroup analyses defined by patients’ pre-diagnosis PA levels revealed that the results were mainly driven by individuals who were sufficiently active before the cancer diagnosis, pointing towards the central role of patients’ self-efficacy beliefs about exercise for the maintenance of PA levels in the presence of structural barriers.

It has long been known that one’s self-efficacy can be influenced by environmental factors [44], potentially because its four main sources (mastery experiences, vicarious experiences, verbal persuasion, and emotional and physiological states) might be shaped by the perception of environmental circumstances [45]. Thus, environmental circumstances, including structural conditions that enable or even encourage individuals to act self-efficiently, seem essential. In the case of PA in people with cancer, these comprise, for instance, the availability of tailored exercise programs and expert contact persons. However, empirical evidence on the relationship between structural barriers and self-efficacy regarding PA in cancer patients is limited. In a study among rural breast cancer survivors, there was a negative association between exercise self-efficacy, barrier self-efficacy, and perceived barriers to PA [31]. A negative relationship was also found in a study among breast cancer patients between barriers and self-efficacy toward those barriers [32]. However, these and other studies that have addressed barriers to exercise in cancer patients have not related them solely to structural barriers but included personal attitudes (e.g., “lack of motivation”) and environmental factors (e.g., “bad weather”) [11,28,46]. The results of this study show that the relationship with PA self-efficacy also becomes evident when considering purely structural barriers.

In contrast, the results of this study did not show a direct association between structural barriers and the intention to exercise, which is, however, consistent with a study that distinguished different types of barriers to PA in cancer patients, i.e., global, practical, and health barriers [47]. Among these, practical barriers—most congruent with the structural barriers in the current study—were not related to intention for PA. Taken together, it is an interesting finding that the intention for PA in cancer patients appears to be independent of the perception of structural barriers. Intention formation may be more strongly determined by social-cognitive factors, i.e., not only self-efficacy but also attitudes or subjective norms toward PA [48], which appear to be relatively independent of structural barriers. In contrast, the construct of PA self-efficacy as such contains a direct reference to perceived barriers and thus seems to be more relevant in this regard [21].

An important aim of this study was to determine whether the discussed social-cognitive factors, i.e., PA self-efficacy and PA intention, can mediate the known association of structural barriers with activity behavior in cancer patients [15,16]. Indeed, we could detect an indirect effect, in which the association was mediated by PA self-efficacy but not by PA intention. Both findings regarding the significant role of self-efficacy and the non-significant role of intention as mediators in explaining PA behavior of cancer patients are consistent with the studies cited above [31,47]. Additionally, we detected a significant mediation consisting of a serial pathway via PA self-efficacy and PA intention. This further strengthens the central role of self-efficacy in helping cancer patients to become or remain sufficiently physically active, whether directly related to exercise behavior or via increased intention. It shows that self-efficacy has the potential to mitigate unfavorable structural conditions. This insight can be used to improve the activity behavior of people with cancer in two ways, either by strengthening patients’ self-efficacy or by reducing structural barriers.

There are numerous behavior change techniques that are targeted at increasing PA self-efficacy [49]. In line with Bandura’s self-efficacy theory, mastery experiences play a central role in gaining self-efficacious beliefs [50]. In this regard, techniques such as action planning [51,52] as well as prompts or graded tasks [52] were revealed as effective in the context of PA. The results of a meta-analysis by Finne et al. indicate that other behavior change techniques may be more effective for increasing PA in cancer populations than in non-clinical populations, such as gradually establishing PA behavior while avoiding social comparisons [53]. Future reviews could identify which specific behavior change techniques are most effective with regard to exercise self-efficacy in cancer patients to determine relevant components for PA interventions in this population.

In attempting to reduce structural barriers, it is essential to consider that cancer patients encounter diverse psychosocial and disease-related challenges after their diagnosis [54]. In a mixed-methods systematic review, Clifford et al. highlighted treatment-related side effects, lack of time, and fatigue as key barriers for cancer patients to engage in PA [12]. We argue that reducing structural barriers can help to reduce these barriers as well. For instance, the provision of information material about exercise during cancer treatment and referral to tailored PA programs have the potential to effectively reduce the various, sometimes specific, concerns about PA [55,56]. This could also strengthen the cancer patients’ self-efficacy to be physically active despite treatment-related side effects. Furthermore, low-threshold exercise programs that can be integrated into the individual’s therapy schedule could save time and resources and may facilitate participation in exercise programs. In this regard, healthcare professionals treating cancer patients, e.g., oncologists, radiation therapists, or oncology nurses, may play an essential role. Through in-depth counseling based on the counseling steps of the 5A framework, including support in setting and achieving PA goals, healthcare professionals can guide patients to become more physically active despite different barriers [39]. In case of uncertainty as to whether a patient can safely exercise without further medical supervision, the option of direct referral from medical specialists to in-house exercise experts, physical therapists, or outpatient rehabilitation professionals could represent an additional effective measure to alleviate structural barriers [57].

The results of this study further highlight the necessity of simultaneously considering structural barriers and self-efficacy for PA, as they seem to be closely related. Consequently, focusing on the concept of barrier self-efficacy [32], particularly on structural barriers, promises valuable insights into how cancer patients can be supported in overcoming structural challenges and achieving sufficient PA levels. Future PA interventions in cancer patients should optimally integrate ways to strengthen patients’ self-efficacy while overcoming barriers with professional guidance. For instance, a planned PA intervention by Millet et al. includes health coaching by an exercise physiologist to support barrier identification and problem solving [58]. Exercise professionals working in community-based exercise centers who are educated on the specific conditions and needs of cancer patients may also serve as PA counselors [59].

This study further revealed that the mediation of structural barriers to the PA behavior of cancer patients did not apply equally to all individuals in our study population but primarily to those who were sufficiently active before the cancer diagnosis. In previous studies, we already showed that different determinants are associated with PA change patterns after cancer diagnosis [9] and that structural barriers were only associated with PA levels among those who met PA guidelines before their diagnosis [16]. Thus, while previous research has emphasized that a cancer diagnosis may act as a teachable moment that should be used to increase PA levels [60], this study highlighted possibilities for how to prevent the risk of losing sufficient pre-diagnosis activity levels. In this regard, our findings suggest that maintaining self-efficacy to engage in PA despite structural barriers plays an essential role. An explanation could be that the group of previously active cancer patients has higher expectations with regard to their PA behavior, also requiring higher self-efficacy beliefs. Perhaps, they have the desire to maintain a certain level or type of activity after the diagnosis, which may be challenging due to physical side effects and/or time constraints of cancer therapy. This interpretation would be in line with a previous finding, revealing that the influence of PA counseling by physicians on PA levels of cancer patients was mediated by the satisfaction with this advice, but only for the subgroup of previously active individuals, who may need in-depth PA guidance [39]. Interestingly, a previous study showed that healthcare professionals seem to preferentially recommend PA to individuals with an apparently low affinity for PA, e.g., with low fitness levels [61]. This study now shows that it is actually the group of (formerly) active patients that requires particular support by healthcare professionals with regard to structural barriers. Thus, PA structures specifically addressing the needs of (formerly) active patients are required, e.g., through counseling opportunities or informational resources that optimally incorporate techniques for increased self-efficacy to overcome potential structural barriers.

To our knowledge, this was the first study to explore the relationship between structural barriers, PA self-efficacy, PA intention, and post-diagnosis PA in a sample of cancer patients, and the results revealed valuable insights. However, some limitations need to be considered when interpreting the findings. As data were assessed as self-reports by cancer patients, they may be biased in the sense of socially desirable answers or recall biases. This could explain the relatively high number of sufficiently active individuals in the present sample as well as high mean values for PA self-efficacy and PA intention. Accordingly, the perception of structural barriers was low on average. Further, although PA self-efficacy and PA intention have been revealed as the strongest social-cognitive determinants of cancer patients’ activity levels, it might be important for future research to also consider other social-cognitive and behavioral factors as potentially relevant for the perception of structural barriers for PA. In this context, it also has to be noted that the cross-sectional design of the current study does not allow causal inferences. Thus, the proposed mechanisms underlying the association between structural barriers and post-diagnosis PA should be validated in longitudinal studies.

## 5. Conclusions

In conclusion, this study suggests that the relationship between structural barriers and PA after the cancer diagnosis is mediated by social-cognitive constructs via different indirect pathways. In this regard, cancer patients’ self-efficacy to be physically active plays a central role in mitigating the perception of structural barriers. The mediation, however, was only found for the group of pre-diagnosis active individuals, which points to the importance of tailoring PA interventions to patients’ previous PA experience. While future research should evaluate additional ways to effectively increase inadequate activity levels in inactive cancer patients, previously active cancer patients appear to benefit from interventions that address the perception of structural barriers. Strategies to directly reduce structural barriers or increase patients’ self-efficacy in dealing with these barriers could thus make a valuable contribution to sustained PA behavior after the cancer diagnosis.

## Figures and Tables

**Table 1:** Descriptive statistics of sample characteristics (*n* = 856).

	Mean or abs. Number	*SD* or %
Age (years) ^a,b^	58.2	12.4
BMI (kg/m²) ^a^	26.3	4.8
Sex		
Female	516	60.3%
Male	314	39.7%
Educational level ^c^		
Lower	445	52.0%
Higher	411	48.0%
Cancer type		
Breast cancer	433	50.6%
Prostate cancer	216	25.2%
Colorectal cancer	207	24.2%
Time since diagnosis (months) ^a,d^	14.8	7.6
Current treatment status		
No treatment	463	55.3%
Receiving treatment	375	44.7%
Chemotherapy ^e^		
No	469	55.3%
Yes	379	44.7%
Radiotherapy ^e^		
No	376	44.3%
Yes	472	55.7%
Hormone therapy ^e^		
No	543	64.6%
Yes	297	35.4%
Co-morbidities		
None	405	47.3%
≥1	451	52.7%
Pre-diagnosis MVPA		
0–149 min/week	317	37.0%
≥150 min/week	539	63.0%
Post-diagnosis MVPA		
0–149 min/week	389	45.4%
≥150 min/week	467	54.6%
5A Score for PA counseling ^a,f^	1.0	0.9
Structural barrier score ^a,g^	0.7	0.7
PA intention ^a,h^	4.9	1.3
PA self-efficacy ^a,i^	4.4	1.7

Notes. *SD*—standard deviation; PA—physical activity; MVPA—moderate-to-vigorous physical activity. ^a^ Displayed as mean (*M*) and standard deviation (*SD*). ^b^ Range: 23–82 years. ^c^ Lower: no degree or (lower-) secondary education degree; Higher: diploma qualifying for university or university degree. ^d^ Range: 0–30 months. ^e^ No: Never having received this treatment; Yes: having received or currently receiving this treatment. ^f^ Weighted sum score for PA counseling based on 5A framework, scale ranging from 0 to 5; higher values indicate more comprehensive counseling. ^g^ Perceived impediment for PA by seven structural barriers, scale ranging from 0 to 3, higher values indicate stronger impediment. ^h^ Intention to perform at least 150 min MVPA/week, scale ranging from 0 to 6; higher values indicate higher intention. ^i^ Confidence to perform at least 150 min MVPA/week, scale ranging from 0 to 6; higher values indicate higher self-efficacy.

**Table 2:** Regression analyses on associations of structural barriers, physical activity self-efficacy, physical activity intention, and sufficient post-diagnosis physical activity within the mediation model.

Predictors	Criterion											
	PA Self-Efficacy ^a^				PA Intention ^a^				Sufficient Post-Diagnosis PA ^b^			
	Coefficient	*SE*	*p*-Value	95% CI	Coefficient	*SE*	*p*-Value	95% CI	Coefficient	*SE*	*p*-Value	95% CI
Structural barrier score ^c^	−0.35	0.09	**<0.001**	(−0.53; −0.17)	−0.03	0.05	0.630	(−0.13; 0.08)	−0.19	0.14	0.171	(−0.47; 0.08)
PA self-efficacy	----	----	----	----	0.55	0.03	**<0.001**	(0.50; 0.60)	0.41	0.08	**<0.001**	(0.25; 0.57)
PA intention	----	----	----	----	----	----	----	----	0.55	0.11	**<0.001**	(0.33; 0.78)
Age	0.02	0.01	**0.004**	(0.01; 0.03)	−0.00	0.00	0.486	(−0.01; 0.00)	−0.01	0.01	0.180	(−0.03; 0.01)
Sex ^d^	0.35	0.23	0.127	(−010; 0.80)	−0.15	0.12	0.221	(−0.39; 0.09)	0.08	0.36	0.439	(−0.42; 0.96)
Educational level	0.42	0.11	**<0.001**	(0.20; 0.63)	0.08	0.06	0.199	(−0.04; 0.20)	0.17	0.18	0.344	(−0.18; 0.53)
BMI	−0.05	0.01	**<0.001**	(−0.07; −0.03)	−0.01	0.01	0.116	(−0.03; 0.00)	−0.02	0.02	0.242	(−0.06; 0.2)
Cancer type												
Prostate cancer ^e^	−0.06	0.26	0.806	(−0.57; 0.44)	0.00	0.14	0.989	(−0.28; 0.28)	−1.26	0.42	**0.003**	(−2.08; −0.44)
Colorectal cancer ^f^	−0.03	0.20	0.897	(−0.41; 0.36)	−0.00	0.14	0.982	(−0.20; 0.20)	−1.30	0.31	**<0.001**	(−1.91; −0.69)
Time since diagnosis	−0.01	0.01	0.322	(−0.02; 0.01)	0.00	0.00	0.893	(−0.01; 0.01)	0.04	0.01	**<0.001**	(0.02; 0.07)
Co-morbidities	−0.27	0.11	**0.015**	(−0.50; −0.05)	0.03	0.07	0.616	(−0.10; 0.16)	−0.29	0.19	0.119	(−0.66; 0.08)
5A score ^g^	0.16	0.06	**0.005**	(0.05; 0.27)	0.06	0.03	**0.034**	(0.00; 0.12)	0.25	0.10	**0.011**	(0.06; 0.44)
Pre-diagnosis PA ^h^	0.91	0.12	**<0.001**	(0.68; 1.14)	0.18	0.07	**0.013**	(0.04, 0.32)	1.47	0.19	**<0.001**	(1.10, 1.84)
Considered section of the mediation model	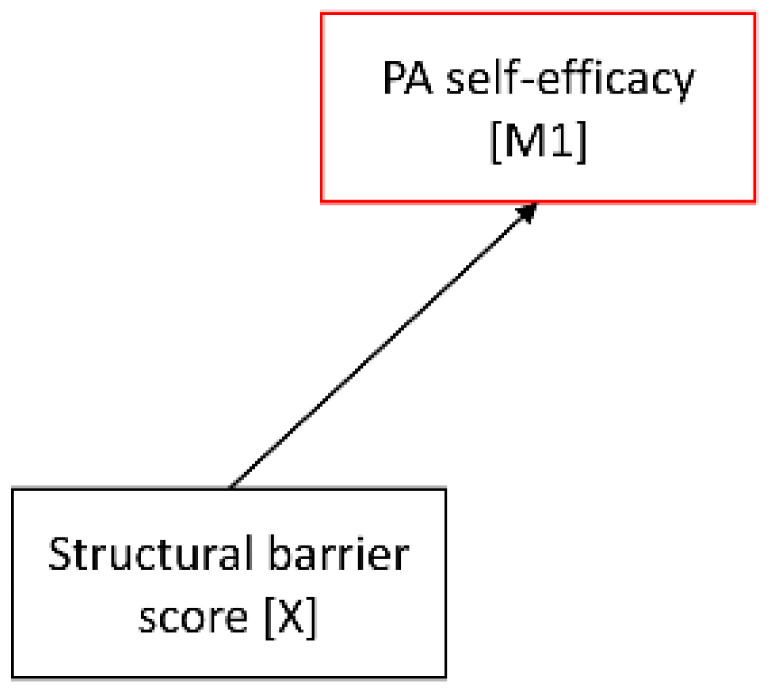				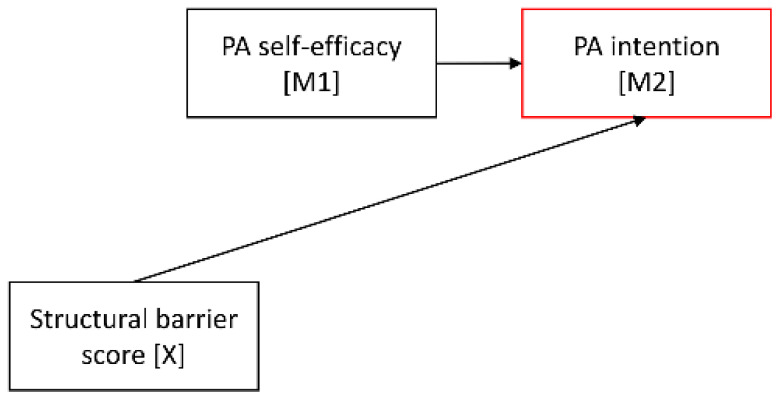				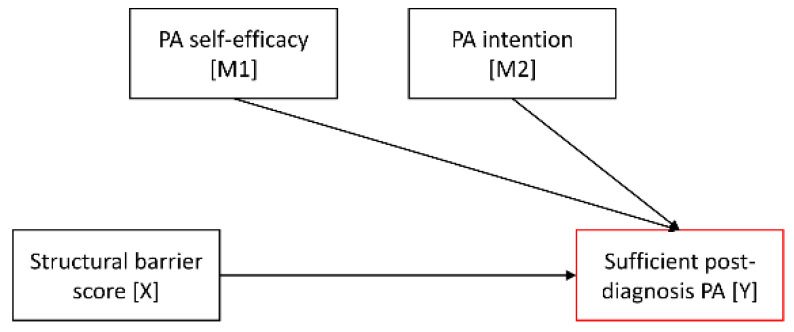			

Notes. *n* = 856. Bold values indicate *p* < 0.05. PA—physical activity; *SE*—standard error; CI—confidence interval. ^a^ Linear regression analysis with continuous outcome variable. A heteroscedasticity-consistent standard estimator was used. ^b^ Logistic regression analysis with binary outcome variable, i.e., meeting PA guidelines of 150 min moderate-to-vigorous PA (MVPA) per week. ^c^ Perceived impediment for PA by seven structural barriers; higher values indicate stronger impediment. ^d^ 0: female; 1: male. ^e^ Cancer type, dummy-coded; 0: breast cancer, colorectal cancer; 1: prostate cancer. ^f^ Cancer type, dummy-coded; 0: breast cancer, prostate cancer; 1: colorectal cancer. ^g^ Weighted sum score for PA counseling based on 5A framework; higher values indicate more comprehensive counseling. ^h^ Sufficient pre-diagnosis PA; 0: not meeting PA guidelines of 150 min MVPA per week; 1: meeting PA guidelines of 150 min MVPA per week.

**Table 3:** Mediation analyses testing the direct and indirect effects of structural barriers on post-diagnosis physical activity via physical activity self-efficacy and physical activity intention in the overall sample and in subsamples divided by pre-diagnosis physical activity.

Analyzed Sample									
Statistical Model	Overall Sample			Previously Sufficiently Active ^a^			Previously Insufficiently Active ^b^		
**Direct Effect**	Effect	*SE*	95% CI	Effect	*SE*	95% CI	Effect	*SE*	95% CI
Structural barrier score ^c^ → Post-diagnosis PA ^d^	−0.19	0.14	(−0.47; 0.08)	−0.16	0.18	(−0.52; 0.19)	−0.18	0.24	(−0.64; 0.29)
**Indirect effect(s) via**	Effect	*Boot SE*	Boot 95% CI	Effect	*Boot SE*	Boot 95% CI	Effect	*Boot SE*	Boot 95% CI
a. PA self-efficacy	**−0.14**	**0.05**	**(−0.25; −0.06)**	**−0.23**	**0.08**	**(−0.41; −0.10)**	−0.05	0.05	(−0.18; 0.02)
b. PA intention	0.01	0.03	(−0.08; 0.04)	0.02	0.03	(−0.04; 0.09)	−0.07	0.07	(−0.24; 0.04)
c. PA self-efficacy and PA intention in serial	**−0.11**	**0.04**	**(−0.19; −0.05)**	**−0.11**	**0.05**	**(−0.22; −0.03)**	−0.07	0.06	(−0.21; 0.03)

Notes. PA—physical activity; *SE*—standard error; CI—confidence interval. All analyses were adjusted for age, sex, educational level, BMI, cancer type, time since diagnosis, co-morbidities, and PA counseling. The mediation analysis in the overall sample was further adjusted for sufficient pre-diagnosis PA. ^a^ Subsample of participants who were meeting PA guidelines of 150 min moderate-to-vigorous PA (MVPA) per week pre-diagnosis (*n* = 539). ^b^ Subsample of participants who were not meeting PA guidelines of 150 min MVPA per week pre-diagnosis. ^c^ Perceived impediment for PA by seven structural barriers; higher values indicate stronger impediment. ^d^ Sufficient post-diagnosis PA, i.e., meeting PA guidelines of 150 min MVPA per week.

## Data Availability

The data that support the findings of this study are available from the corresponding author upon reasonable request.
